# Transcriptome-Based Analysis of the Mechanism of Acute Manganese-Induced Immune Function Decline and Metabolic Disorders in Estuarine Tapertail Anchovy (*Coilia nasus*)

**DOI:** 10.3390/ani16060974

**Published:** 2026-03-20

**Authors:** Xiaolu Shen, Yongli Wang, Mingchun Ren, Dongyu Huang, Jiaze Gu, Leimin Zhang, Hualiang Liang, Xiaoru Chen

**Affiliations:** 1 College of Fishes and Life Science, Shanghai Ocean University, Shanghai 201306, China; 2 Tongwei Agricultural Development Co., Ltd., Key Laboratory of Nutrition and Healthy Culture of Aquatic Livestock and Poultry, Ministry of Agriculture and Rural Affairs, Healthy Aquaculture Key Laboratory of Sichuan Province, Chengdu 610093, China; 3 Key Laboratory of Integrated Rice-Fish Farming Ecology, Ministry of Agriculture and Rural Affairs, Freshwater Fisheries Research Center, Chinese Academy of Fishery Sciences, Wuxi 214081, China; 4 Wuxi Fisheries College, Nanjing Agricultural University, Wuxi 214081, China

**Keywords:** acute manganese stress, immune disorders, metabolic disorders, oxidative stress, transcriptome analysis, *Coilia nasus*

## Abstract

To characterize the transcriptional and physiological alterations induced by manganese stress in *Coilia nasus*, juveniles (mean weight 5.0 ± 0.2 g) were subjected to either manganese exposure (5.50 ± 0.03 mg/L) or control (0 mg/L) for a 12 h period. Subsequently, gill tissues were excised for evaluation of antioxidant parameters and RNA-Seq analysis. A total of 753 DEGs were identified in the manganese exposure group compared to controls, comprising 287 up-regulated and 466 down-regulated genes. GO and KEGG enrichment analysis of DEGs showed that most of the DEGs were involved in immune and metabolic pathways, which disturbed the biological processes related to immunity and metabolism at the molecular level. The acute manganese stress initiated a multi-level antioxidant response to cope with oxidative stress in *Coilia nasus*. This finding was further supported by the significant increase in MDA content and significant decrease in GSH content and GSH-Px activity under manganese exposure, while SOD and CAT activities were significantly increased. Simultaneously, the acute manganese stress triggered profound metabolic reprogramming to cope with energy pressure in *Coilia nasus*, which showed that manganese exposure significantly down-regulated energy metabolism-related genes (*pfkm*, *pgam2*, *eno3*, *pkm*, *aqp9*, *apoa1*, *tkt*, *sds*); furthermore, the overall energy metabolism network was widely inhibited, while lipid metabolism-related genes (*fabp3*, *cpt1a*) were significantly up-regulated to compensatorily activate fatty acid transport and β-oxidation pathways. In addition, the acute manganese stress initiated a complex immune response pattern to cope with cell damage in *Coilia nasus*, which showed that manganese exposure significantly enhanced the expression of inflammatory signaling genes (*mapk1*, *stat1*, *tgfb3*); furthermore, certain inflammatory pathways were activated, while the expressions of immune regulatory genes (*traf6*, *il-10*) were significantly decreased. In summary, these results indicated that manganese exposure could impair immune function, disrupt metabolism, and induce oxidative stress in *Coilia nasus*.

## Simple Summary

To reveal the effects of manganese stress on gene expression, molecular pathways and physiological functions of *Coilia nasus*, two libraries of a manganese treatment group (Mn) and control group (C) were constructed. Illumina sequencing technology was used for transcriptome sequencing, and antioxidant-related indicators were also measured. The main point of this study showed that Mn could cause oxidative stress damage to the body and inhibit immune function.

## 1. Introduction

In recent years, industry has developed rapidly, and wastewater from industries such as mining, metallurgy, and electroplating contains high concentrations of manganese [1]. In agricultural production activities, manganese is often used as a trace element supplement or as an effective component of commonly used disinfectants [2]. The manganese produced by these practices will eventually circulate to the water, resulting in an increase in manganese content in the water, which in turn affects the physical health of aquatic organisms.

An appropriate amount of manganese in water has a positive effect on the growth and metabolism of aquatic organisms; however, high concentrations of manganese ions can damage the gill tissue of fish, leading to respiratory disorders, anorexia and growth stagnation. Long-term exposure may also cause nervous system damage [3]. A large number of studies have shown that the toxicity of manganese to fish is concentration-dependent and time-dependent, and varies with fish species, developmental stage, and water physicochemical properties. Under chronic exposure conditions, manganese can cause significant sublethal effects on fish even at relatively low environmental relevant concentrations. A study found that when juvenile groupers (*Epinephelus moara*♀ × *E. lanceolatus*♂) were exposed to 0.5 to 4 mg/L Mn^2+^ solution for 30 days, the liver showed obvious oxidative stress and apoptosis [4]. In addition, manganese can also interfere with the immune function and endocrine system of fish. For example, studies in red sea bream (*Pagrus major*) and black rockfish (*Sebastes schlegelii*) showed that the complement activity and lysozyme activity of blood were significantly reduced after 14 days of exposure to 2 mg/L manganese, indicating that their non-specific immunity was inhibited [5,6]. For the early life stage of fish, the toxicity of manganese is particularly prominent. Marine medaka (*Oryzias melastigma*) embryos showed a series of developmental toxicity symptoms such as increased heart rate, delayed hatching, decreased hatching rate and increased deformity rate after exposure to different concentrations of MnCl_2_. These effects are closely related to the increase in oxidative stress level and the activation of inflammatory response in embryos [7]. Similarly, a study on *Astyanax lacustris* also confirmed that as exposure time extended from 96 h to 21 days, the accumulation of manganese in the gills and liver continued to increase, leading to changes in the activity of glutathione S-transferase (GST) and other detoxification enzymes [8]. In addition, manganese can also accumulate in organisms through the food chain, and long-term exceedance of safety standards in aquatic products may affect their food safety, thereby endangering human health [9,10]. Therefore, it is of great significance to study the toxicity of manganese in water to aquatic organisms.

Gills are the core respiratory and excretory organs of fish, and are also involved in osmoregulation, ammonia nitrogen excretion and ion balance. When heavy metals (such as cadmium, copper, lead, zinc, manganese, etc.) exceed permissible levels in the water, the gills, as the primary organ in direct contact with the external environment, bear the brunt of the toxic effects, which leads to physiological dysfunction and even death of fish [11]. In addition, the gill mucosal layer contains mucus cells that secrete mucins and antimicrobial peptides, forming a chemical and physical barrier that helps resist pathogen invasion and provides immune functions [12]. Hedayati et al. found that high concentrations of manganese exposure could cause gill damage in Caspian carp (*Rutilus caspicus*), including epithelial bulge, hyperplasia, gill lamellae fusion, aneurysm formation and gill lamellae curl [13]. Maria et al. also found that short-term exposure to high concentrations of manganese in water could cause peroxidation damage to the gills of goldfish (*Carassius auratus*) and trigger organ-specific antioxidant responses involving differential regulation of superoxide dismutase (SOD), catalase (CAT) and glutathione peroxidase (GSH-Px) activities [14]. Therefore, fish gills are very sensitive to pollutants such as heavy metals, and changes in their tissue structure and physiological functions can be used as early biomarkers to evaluate the degree of water environmental pollution and biological toxicity effects.

Estuarine Tapertail Anchovy (*Coilia nasus*), renowned as the foremost delicacy among the ‘Three Delicacies of the Yangtze River’, has high nutritional value. It is rich in high-quality protein, docosahexaenoic acid, eicosapentaenoic acid and a variety of minerals [15]. It is a high-end aquatic product loved by consumers. However, due to the depletion of fishery resources, including wild *Coilia nasus*, the Yangtze River has implemented a comprehensive fishing ban. Therefore, the development of artificial breeding of *Coilia nasus* has significant economic potential and offers social benefits. *Coilia nasus* aquaculture has extremely high requirements on water quality, so the related aquaculture industry is also facing significant challenges, especially the sensitivity to trace elements such as manganese, which is one of the core challenges faced by its aquaculture industry [16]. Therefore, in-depth study of the specific damage threshold and mechanism of manganese excess on the growth, reproduction, immunity, stress and other physiological functions of *Coilia nasus* can provide an accurate scientific basis for aquaculture water quality standards, feed safety and aquaculture environment management, avoid huge economic losses, and support the sustainable development of aquaculture. At present, there is a lack of direct research on the effect of manganese stress on *Coilia nasus*. In this study, we diluted the concentration of manganese ions in water to 5.50 ± 0.03 mg/L according to the preliminary experimental results, and continued to stress for 12 h. This concentration selection can not only ensure that a strong and detectable toxic signal can be generated in a short time, but also avoid rapid death caused by high concentration, thus providing an ideal window for observing early molecular and cellular stress responses. The purpose of this study was to reveal the effects of manganese stress on gene expression, molecular pathways and physiological functions in the gills of *Coilia nasus* by combining antioxidant index detection and transcriptome sequencing analysis, and to provide new ideas and directions for the healthy breeding of *Coilia nasus*.

## 2. Materials and Methods

### 2.1. Experimental Materials and Fish

Healthy juvenile *Coilia nasus* were provided by Yangzhong Base of Freshwater Fishery Research Center, Chinese Academy of Fishery Sciences (Zhenjiang, China). They were fed with commercial feed during the temporary culture period.

The MnCl_2_·4H_2_O used in the experiment was analytically pure. In preliminary experiment, we set up a control group and 6 treatment groups. The concentrations of the 7 groups were 0 mg/L (control group), 1.35 ± 0.03 mg/L (PD1), 2.69 ± 0.06 mg/L (PD2), 4.15 ± 0.04 mg/L (PD3), 5.50 ± 0.03 mg/L (PD4), 6.90 ± 0.05 mg/L (PD5) and 8.31 ± 0.04 mg/L (PD6), respectively. The number of dead fish was recorded at 0 h, 4 h, 8 h, 12 h, 16 h, 20 h and 24 h, and the mortality rate was calculated to determine the concentration and duration of acute stress (Table S1). Based on the semi-lethal data from the preliminary experiment, the manganese ions in the water were diluted to a predetermined concentration (5.50 ± 0.03 mg/L) and stress duration (12 h). The experimental water was purified water after 3 days of aeration. The water quality indexes were as follows: pH value was 7.2, dissolved oxygen was greater than 7.0 mg/L, and water temperature was 25.0 ± 0.5 °C.

### 2.2. Management Methods

The juvenile *Coilia nasus* (average weight 5.0 ± 0.2 g) were randomly distributed into six indoor recirculating aquaculture tanks and assigned to two experimental groups: a manganese exposure group (Mn) and a control group (C). Each group consisted of three tanks, with 20 fish per tank. The behavior of the fish was observed throughout the experiment. After 12 h, three fish were randomly selected from each tank and anesthetized with MS-222 (0.2 g/L). Gill tissue samples were collected from three fish in each tank. From each fish, the gills were divided into two portions: one portion was used for enzyme activity assay and the other for transcriptome sequencing analysis. For transcriptome analysis, the gill tissue from the three fish in the same tank was pooled into a single sample. All samples were stored at −80 °C for subsequent testing. In order to prevent the influence of feeding, no feed was given during the experiment.

### 2.3. Determination of Antioxidant-Related Parameters

The gill tissue sample of 0.1 g was placed in a 2 mL centrifuge tube. Two clean steel beads were added, followed by 0.9 mL of normal saline, and the mixture was thoroughly homogenized using a homogenizer. After homogenization, the mixture was centrifuged at 4 °C, 4000 rpm/min for 10 min. The resulting supernatant was collected, transferred to a clean 1.5 mL centrifuge tube, and kept on ice until analysis. The levels of antioxidant-related parameters, including SOD, CAT, malondialdehyde (MDA), glutathione (GSH) and GSH-Px, were measured using kits from Nanjing Jiancheng Bioengineering Institute (Nanjing, China). The succinct methods of determining antioxidant-related indicators are shown in Table 1. The specific methods are as follows: (1) The activity of SOD (U/mg prot) was detected by the WST-1 colorimetric method (A001-3-2), and the amount of enzyme required to inhibit 50% NBT photochemical reduction rate per mg protein in the reaction system was measured as 1 unit. (2) CAT activity (U/mg prot) was measured by the ammonium molybdate colorimetric method (A007-1-1). One unit of enzyme activity was defined as the decomposition of 1 μmol H_2_O_2_ per second per milligram of protein. (3) The content of MDA (nmol/mg prot) was determined by the thiobarbituric acid (TBA) method (A003-1-2), and the absorbance of red adduct at 532 nm was measured. (4) The content of GSH (μmol/g prot) was determined by the DTNB colorimetric method (A006-2-1) at 405 nm. (5) GSH-Px activity (U/mg prot) was measured by the colorimetric method (A005-1-2), which was defined as the amount of enzyme corresponding to the reduction in GSH consumption by 1 μmol/L per minute per milligram of protein at 37 °C (excluding the background of non-enzymatic reaction). All enzymatic assays were performed using the Coomassie brilliant blue method (A045-2-2, according to GB/T 23570-2009 [17]) to simultaneously determine the tissue protein concentration (mg prot/mL) as the enzyme activity normalization benchmark. All tests were performed on a Thermo Fisher Multiskan GO full-wavelength microplate reader (Shanghai, China), and the operation process strictly followed the instructions of each kit. The data obtained were analyzed by SPSS 19.0 software. The results are expressed as mean ± standard deviation. The independent-sample t-test was used to test the difference between different groups. Before statistical analysis, all data were evaluated for normality using the homogeneity of variance test. *p* < 0.05 was considered statistically significant.

### 2.4. RNA Extraction, Library Construction, and Sequencing

Total RNA was extracted from the gill tissue samples of the C group and the Mn group using the TRIzol reagent kit (Invitrogen, Carlsbad, CA, USA). RNA quality was validated using an Agilent 2100 Bioanalyzer (Agilent Technology Co., Ltd., Santa Clara, CA, USA). After extraction, eukaryotic mRNA was purified by affinity enrichment with Oligo (dT) magnetic beads (Thermo Fisher Scientific, Waltham, MA, USA). The enriched mRNA was fragmented using a fragmentation buffer, and a sequencing library was generated following stringent quality control. The cDNA library was subsequently amplified by polymerase chain reaction (PCR), and sequencing was performed on an Illumina NovaSeq 6000 platform (Illumina, San Diego, CA, USA) by Kidio Biotechnology Co., Ltd. (Guangzhou, China).

### 2.5. Bioinformatics Analysis

First, we used fastp (v0.18.0) [18] for quality control to filter out low-quality data and obtain clean reads. After the data filtering, the data quality was visually displayed. Next, we used HISAT2 (v2.1.0) [19] software to perform comparative analysis based on the reference genome. Based on the comparison results, the transcript was reconstructed using Stringtie (v3.0.0) [20], and then the gene expression was calculated using RSEM (v1.3.3) [21]. After that, in order to exclude abnormal samples, the reproducibility of samples was determined by Pearson correlation analysis.

### 2.6. Differentially Expressed Genes (DEGs)

In order to obtain a reliable and robust list of DEGs, a multi-algorithm consensus strategy was adopted. The differential expression of RNA between the two groups was analyzed using DESeq2 (v1.20.0) [22] and edgeR (v3.32.1) [23] software. The genes identified as significantly differentially expressed by the above two software packages were defined as a high-confidence differential gene set (the false discovery rate < 0.05 and |log2(fc)| ≥ 1) for all subsequent functional enrichment and mechanism analysis.

### 2.7. Gene Ontology (GO) and Kyoto Encyclopedia of Genes and Genomes (KEGG) Enrichment Analysis

GO enrichment analysis was conducted by mapping DEGs to terms in the GO database (http://www.geneontology.org/, accessed on 5 June 2023) and calculating the number of genes associated with each term. KEGG pathway analysis was also performed to identify enriched pathways. Significantly enriched GO terms and KEGG pathways were identified using a hypergeometric test compared to the background genome.

### 2.8. The Protein–Protein Interaction (PPI) Network Analysis

A PPI network of the DEGs was constructed using the STRING database (http://string-db.org) [24]. Briefly, target gene sequences were aligned to reference protein sequences via Blastx, and interactions were mapped based on homology. The network was then visualized with Cytoscape (v3.10.4) [25].

### 2.9. Real-Time PCR (qRT-PCR) Validation Analysis for DEGs

Validation of the RNA-Seq results was accomplished via qRT-PCR analysis of fifteen selected DEGs. Gene expression levels were normalized to β-actin (forward: AACGGATCCGGTATGTGCAAAGC; reverse: GGGTCAGGATACCTCTCTCTCTCTTGCTCTG) [26]. Total RNA isolated from gill tissues of control and manganese-exposed individuals was used as a template, and amplification was performed on a CFX96 real-time PCR instrument (Bio-Rad, Shanghai, China) employing the HiScript^®^ II One-Step qRT-PCR SYBR Green kit (Vazyme Biotech Co., Ltd., Nanjing, China) in accordance with the manufacturer’s guidelines. Relative quantification was achieved using the standard curve method [27], with primer details provided in Table 2.

## 3. Results

### 3.1. Effects of Manganese Stress on Antioxidant Capacity of Gill Tissue of Coilia nasus

**Figure 1:**
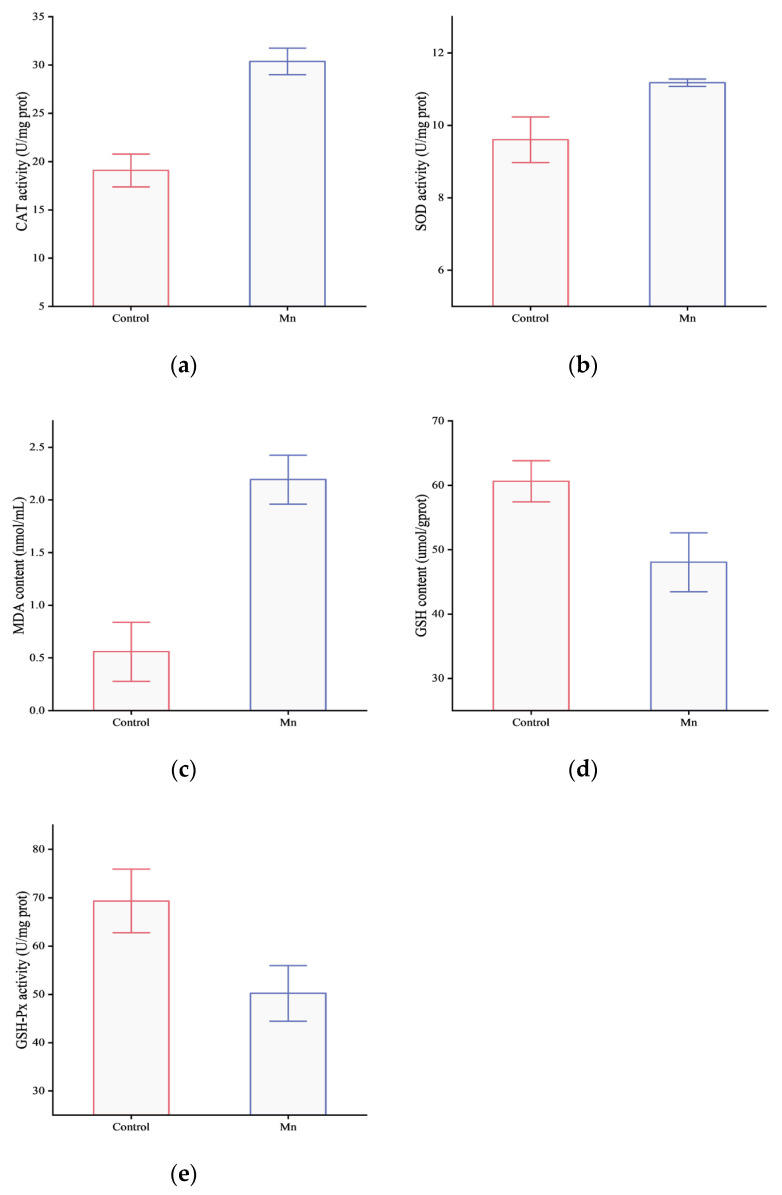
Effects of manganese stress on antioxidant capacity of *Coilia nasus*: (**a**) catalase (CAT); (**b**) superoxide dismutase (SOD); (**c**) malondialdehyde (MDA); (**d**) glutathione (GSH); (**e**) glutathioneperoxidase (GSH-Px). Data are expressed as a graphical representation of the mean and standard deviation bars (means ± S.D.).

As shown in Figure 1, manganese exposure significantly increased MDA content, SOD activity, and CAT activity, but decreased GSH content and GSH-Px activity, compared to controls (*p* < 0.05).

### 3.2. Sequencing Quality and Sequence Comparison Reference Statistics

**Figure 2:**
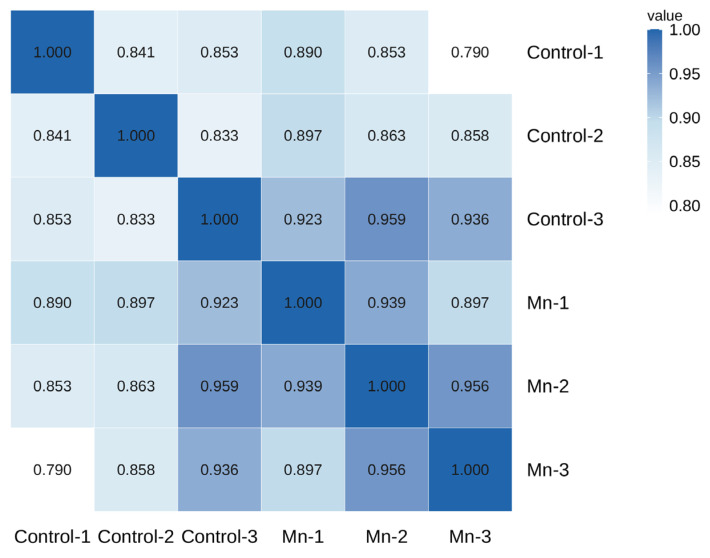
Sample correlation heat map.

A total of 267,322,400 raw reads were generated by transcriptome sequencing of six gill samples (Table 3). After removing low-quality data, 40.5–51.43 million clean reads were retained for each sample, and the Q20 and Q30 values were higher than 97.39% and 93.14%, respectively. The average GC content of all samples was 48.87%. The percentage of uniquely mapped reads ranged from 79.18% to 83.02%, while multi-mapped reads accounted for 2.84% to 3.97% of the total. Sample correlation analysis, visualized as a heat map (Figure 2), demonstrated good intra-group reproducibility, with Pearson correlation coefficients exceeding 0.8 among replicates within both the control and manganese exposure group. Inter-group correlations were also strong, with the lowest coefficient (0.79) observed between Mn-3 and Control-1. These findings confirm the high quality and reliability of the transcriptome data for subsequent analyses.

### 3.3. DEGs

**Figure 3:**
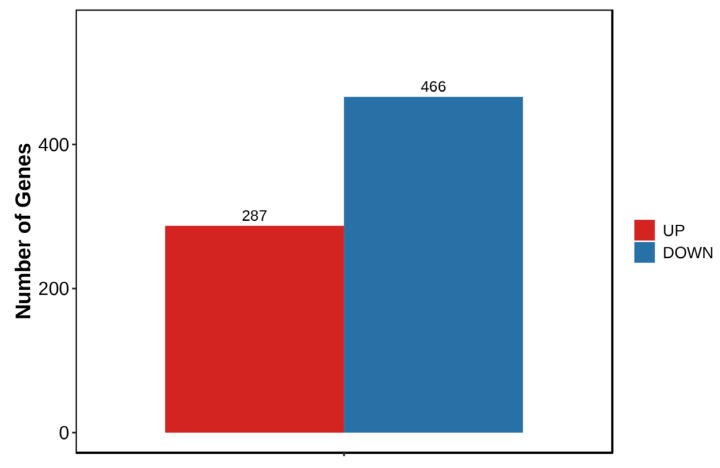
Differential gene statistics.

**Figure 4:**
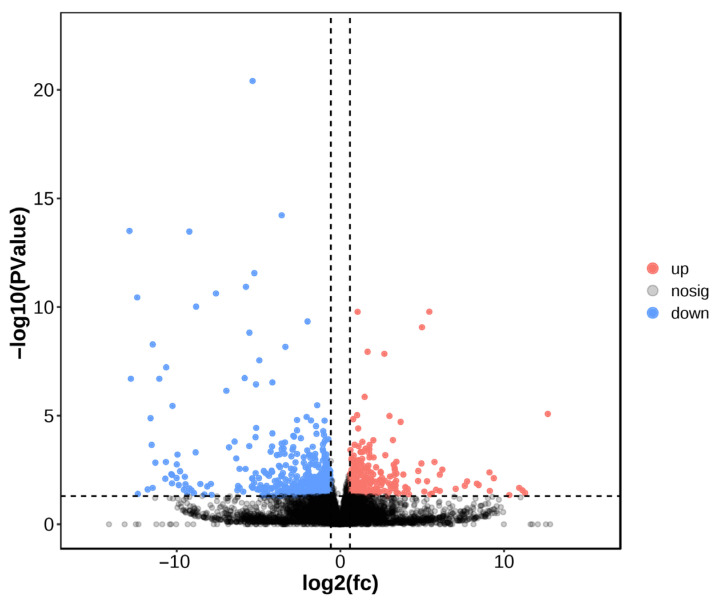
Comparison between C and Mn groups’ volcano plots.

A total of 753 DEGs were identified in the manganese exposure group compared to controls, comprising 287 up-regulated and 466 down-regulated genes (Figure 3). Volcano plot visualization showed that genes at the extremes of the plot exhibited the most pronounced expression differences (Figure 4).

### 3.4. GO and KEGG Pathway Enrichment Analysis

**Figure 5:**
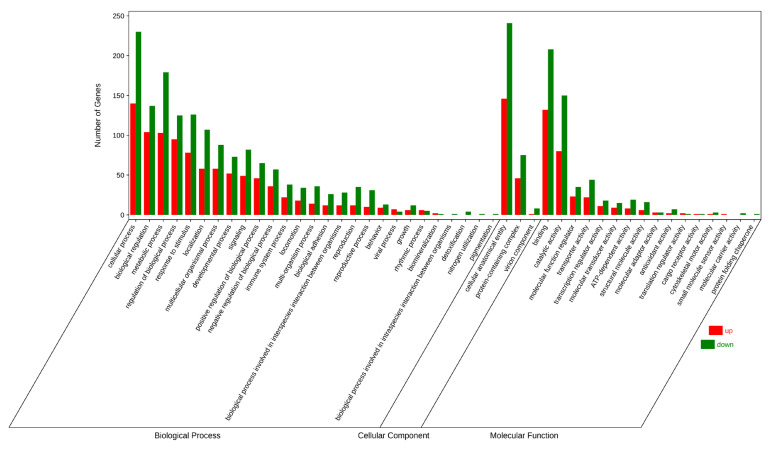
GO enrichment classification histogram.

The identified DEGs were subjected to GO enrichment analysis, and the annotations were categorized into three functional groups: biological processes (BPs), cellular components (CCs), and molecular functions (MFs) (Figure 5). In the biological process category, the main categories were the cellular process, biological regulation and metabolic process. In terms of CCs, the main categories were the cellular anatomical entity and protein-containing complex. In molecular function, the main categories were binding and catalytic activity.

**Figure 6:**
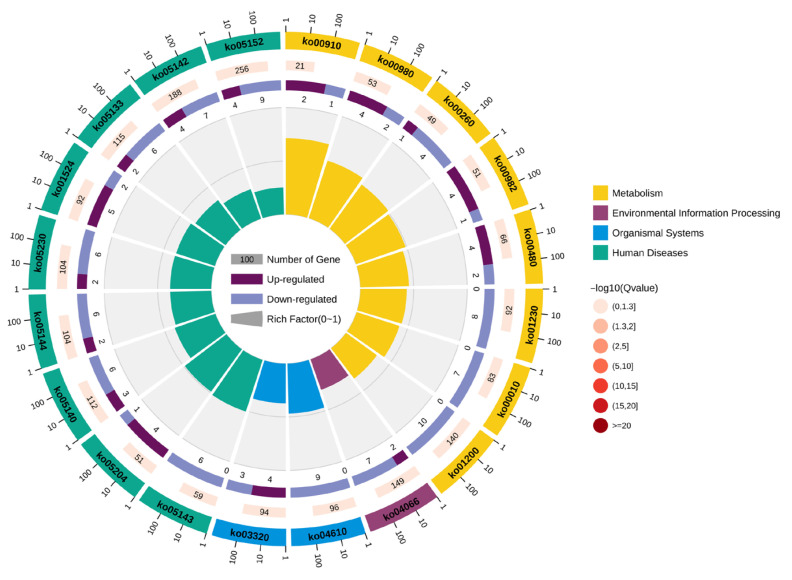
KEGG enrichment cycle diagram.

**Figure 7:**
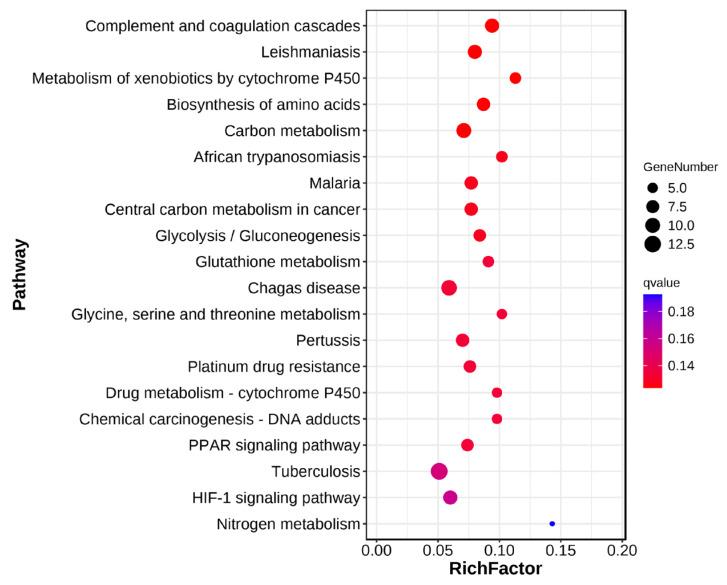
KEGG enrichment bubble diagram.

Based on KEGG functional annotation (*p* < 0.05), significant enrichment was observed for 20 pathways (Figure 6). DEGs in the manganese-exposed group were enriched in complement and coagulation cascades, amino acid biosynthesis, carbon metabolism, glycolysis/gluconeogenesis, and glutathione metabolism compared to controls (Figure 7).

### 3.5. Analysis of Differential Gene Protein Interaction Network

**Figure 8:**
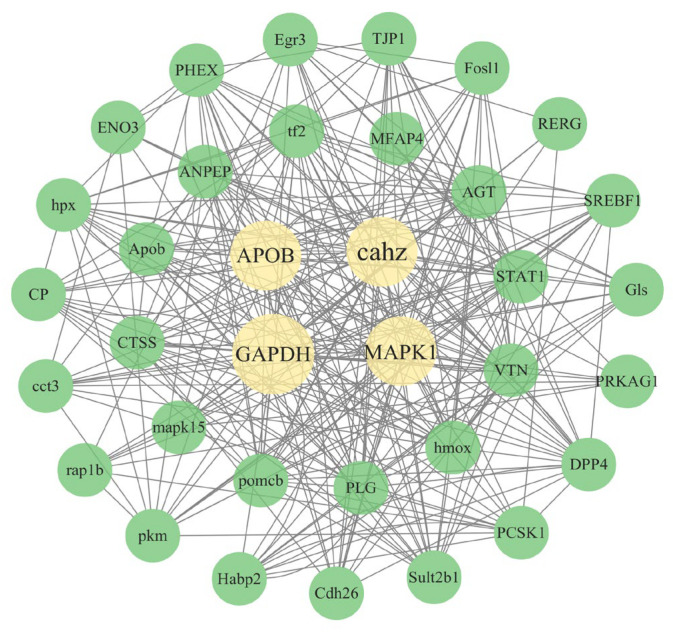
Protein interaction network diagram.

The PPI network analysis was performed to identify key regulatory pathways involved in the response to manganese exposure. As shown in Figure 8, the PPI network showed the interaction between core proteins including APOB, cahz, GAPDH and MAPK1. These proteins play important physiological functions in organisms, and their expression levels or interaction changes may be closely related to the physiological response caused by manganese stress.

### 3.6. Validation of RNA-Seq Results with qRT-RCR

**Figure 9:**
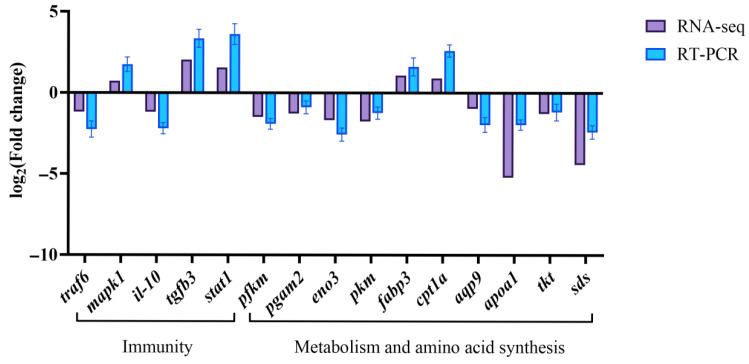
Validation of RNA-Seq results using RT-PCR. The transcriptional expression level of the selected genes was standardized based on the expression level of *β-actin*.

To validate the RNA-Seq results, qRT-PCR was performed to validate the transcriptome data, using fifteen DEGs involved in immunity, metabolism, and amino acid synthesis (*traf6*, *mapk1*, *il-10*, *tgfb3*, *stat1*, *pfkm*, *pgam2*, *eno3*, *pkm*, *fabp3*, *cpt1a*, *aqp9*, *apoa1*, *tkt*, *sds*), and the relative expression levels of fifteen genes caused by manganese stress were quantitatively analyzed by the standard curve method (expressed as mean ± standard deviation) (Figure 9). The expression patterns of the fifteen selected genes were consistent with the RNA-Seq data, confirming the reliability of the transcriptome sequencing results.

## 4. Discussion

### 4.1. Effects of Manganese Stress on Antioxidant Capacity of Gill Tissue of Coilia nasus

Oxidative stress is a state of cell damage caused by the imbalance between reactive oxygen species (ROS) production and antioxidant defense systems. Excessive ROS could cause damage to lipids, proteins and DNA in cells [14]. Studies have shown that exposure to excessive manganese could significantly induce oxidative stress in aquatic animals [16]. *Carassius auratus* were exposed to 1 mM manganese water for 96 h, and the levels of lipid peroxidation products (MDA) in the gill, kidney, liver and brain were significantly increased [14]. Lipid peroxidation is an important marker of damage caused by ROS attacking cell membrane lipids [28]. This indicated that manganese-induced oxidative stress directly damaged the cell membrane integrity of fish [14]. In this study, manganese stress significantly increased MDA content of gill in *Coilia nasus*, indicating that oxidative damage has occurred, and the integrity, fluidity and function (such as ion channels) of the cell membrane have been destroyed. The gill was directly in contact with the water environment, and its membrane structure damage would seriously affect the respiratory and osmotic pressure regulation functions of fish.

In response to oxidative stress, aquatic animals activate the endogenous antioxidant defense system. Among them, the role of SOD is to scavenge superoxide radicals (O_2_^−^) by converting them to H_2_O_2_ and O_2_, while CAT is responsible for the breakdown of hydrogen peroxide into non-toxic H_2_O and O_2_ [29]. In this study, manganese stress led to a significant increase in SOD and CAT activities, indicating that the body was actively initiating its endogenous antioxidant defense mechanism to remove excessive reactive oxygen species. Gabriel et al. found that the activities of SOD and CAT in the kidney, gill, brain and liver of juvenile tambaqui (*Colossoma macropomum*) increased after exposure to manganese ions [30]. Similarly, the activities of SOD and CAT in the liver of juvenile grouper (*Epinephelus coioides*) were also significantly increased in oxidative stress caused by salinity changes [28].

Through its sulfhydryl moiety, GSH directly eliminates free radicals. Moreover, under the catalysis of GSH-Px, it participates in the conversion of hydrogen peroxide and organic peroxides to water and alcohol, thereby safeguarding cells against oxidative injury [30]. GSH-Px is a selenium-dependent enzyme that plays a key role in antioxidant defense [31]. In the present study, diminished levels of GSH and reduced activity of GSH-Px in the gill tissue signified antioxidant depletion and a compromised antioxidant defense system. Under high-concentration manganese stress, the body produces a large amount of ROS, and GSH is consumed in large quantities to directly scavenge free radicals or participate in the reduction of peroxides as a substrate of GSH-Px, resulting in a decrease in its internal reserves [32]. At the same time, high concentrations of manganese may directly or indirectly inhibit the activity of GSH-Px, or under long-term high-intensity oxidative stress, the enzyme itself is inactivated by oxidative damage, resulting in a decrease in its function [31]. Similar defense mechanisms are also reflected in other stressor-related studies. For example, compared with the control group, zebrafish (*Danio rerio*) exposed to 50 μM aluminum trichloride (AlCl_3_) showed a significant decrease in GSH levels, as reported by Capriello et al. [33]. Kim et al. also found that the level of GSH in *Sebastes schlegelii* decreased significantly after long-term exposure to lead [34].

Therefore, when *Coilia nasus* was exposed to high concentrations of manganese, excessive manganese ions (Mn^2+^) accumulated in gill cells, which led to the increase in ROS. ROS attacked polyunsaturated fatty acids in the cell membrane, triggering lipid peroxidation, and generated lipid peroxidation products such as MDA. In order to cope with manganese-induced oxidative stress, the body activated its endogenous antioxidant defense system to remove excessive ROS and repair damage. However, this antioxidant defense response was limited [28]. When the concentration or exposure time of manganese exceeds the tolerance of aquatic animals, the antioxidant enzyme system may be depleted or its activity can be inhibited, resulting in severe oxidative stress damage.

### 4.2. Effects of Manganese Stress on Immune Function of Coilia nasus

Manganese is one of the essential trace elements for organisms, but excessive manganese exposure could significantly affect the physiological functions of aquatic organisms, especially the immune system [35,36]. The immune system of aquatic animals undergoes complex molecular and physiological changes in response to environmental stresses [37,38]. The mitogen-activated protein kinase (MAPK) signaling pathway makes an essential contribution to cell response to external stimuli, regulation of cell proliferation, differentiation, apoptosis and immune response [39]. In this study, manganese stress up-regulated the expression of *mapk1* in gill tissue of *Coilia nasus*, indicating that cells may be activating stress response mechanisms to cope with manganese-induced cell damage and immune challenges. It was found that after 30 days of exposure to Mn^2+^ (0–4 mg/L) in *Epinephelus moara♀× E. lanceolatus♂*, the key molecules p38 and JNK in the MAPK pathway were phosphorylated and activated, indicating that manganese-induced oxidative damage triggers the apoptosis process through the MAPK cascade, which was consistent with the study [40]. As a major anti-inflammatory cytokine, *il-10* helps regulate immune responses by suppressing overactivation, ensuring immune stability, and protecting against tissue injury [41,42]. Under manganese stress, the expression of *il-10* in the gill tissue of *Coilia nasus* decreased, indicating that the body’s anti-inflammatory mechanism may be inhibited, and the pro-inflammatory response dominates. *Stat1* is a key transcription factor in the interferon (IFN) signaling pathway, which could enhance the pro-inflammatory immune response [43,44]. Studies have found that the expression of *stat1* variants in rock bream (*Oplegnathus fasciatus*) is transcriptionally regulated after pathogen stress and tissue damage [45]. The up-regulation of *stat1* in *Coilia nasus* under manganese stress may mean that the body enhances the immune defense against environmental toxins by activating the interferon signaling pathway, and clearing damaged cells or potential pathogens by promoting the inflammatory response. Members of the transforming growth factor-β (TGF-β) family have a dual role in immune regulation, which could promote inflammation and inhibit inflammation, depending on cell type and microenvironment [46]. As one of the isoforms, the up-regulated expression of *tgfb3* under manganese stress may indicate that the body tries to limit the inflammation and damage caused by manganese stress by activating immunosuppression and tissue repair mechanisms. *Traf6* is involved in inflammatory response, cell survival and immune cell development by mediating the triggering of NF-κB and MAPK pathways [47,48]. The expression of *traf6* gene in Pelteobagrus fulvidraco was significantly up-regulated after *Aeromonas hydrophila* infection, indicating its key role in immune response [49]. However, in this experiment, the expression of *traf6* in the gill tissue of *Coilia nasus* decreased under manganese stress, which indicated that manganese stress may pose a serious threat to *Coilia nasus*, and its immune function may also be weakened. Collectively, manganese stress may inhibit key immune hubs by activating stress-induced apoptosis pathways, inhibiting anti-inflammatory regulation, and enhancing pro-inflammatory signals, resulting in the destruction of immune balance.

### 4.3. Effects of Manganese Stress on Lipid Metabolism in Gill of Coilia nasus

*Fabp3* facilitates the intracellular uptake and trafficking of fatty acids [50], which are then transported into mitochondria by *Cpt1a*, the rate-limiting enzyme for long-chain fatty acid oxidation (FAO), β-oxidation and energy production [51,52]. Under manganese stress, the up-regulation of *fabp3* and *cpt1a* expression suggests that the body may face energy supply pressure. When exposed to excess manganese ions, gill epithelial cells may increase ATP consumption due to oxidative stress, or mitochondrial dysfunction leads to decreased energy production efficiency, thereby compensating for the energy gap by enhancing fatty acid metabolic pathways. *Aqp9* is a water–glycerol channel protein that could transport small molecules such as glycerol and lactic acid [53,54]. *Apoa1* facilitates reverse cholesterol transport and regulates lipid metabolism [55,56]. Under manganese stress, the decreased expression of *aqp9* and *apoa1* in the gill tissue of *Coilia nasus* suggests that the lipid biosynthesis process may be reduced, resulting in limited lipid transport, which may be the result of lipid metabolism disorder caused by manganese toxicity. Wang et al. found that the expression of lipid metabolism-related genes in the liver was abnormal and the lipid metabolism pathway was inhibited in the study of *Epinephelus moara♀× E. lanceolatus♂* exposed to Mn^2+^ for a long time, which might represent the eventual outcome of this compensatory failure [40]. In summary, we concluded that the effect of manganese stress on lipid metabolism in fish was a multi-level and multi-target complex process: the initial performance was energy compensatory enhancement, accompanied by decreased lipid synthesis and transport capacity, and long-term exposure led to the overall inhibition of liver lipid metabolism pathways, which may eventually lead to ectopic lipid deposition, energy depletion and organ dysfunction. However, this is only a conjecture and has no data support. Long-term stress experiments are still needed to be designed for further exploration in the future.

### 4.4. Effects of Manganese Stress on Glycolysis and Amino Acid Synthesis in Gill Tissue of Coilia nasus

*Eno3*, *pfkm* and *pgam2* are core glycolytic enzymes. *Pfkm* catalyzes the conversion of fructose-6-phosphate to fructose-1,6-diphosphate, which is the main rate-limiting step of glycolysis. *Pgam2* is responsible for the conversion of 3-phosphoglycerate to 2-phosphoglycerate, and *eno3* catalyzes the dehydration of 2-phosphoglycerate to produce phosphoenolpyruvate, which together maintain the efficient production of cellular ATP and the carbon skeleton [57,58,59]. In the present results, the decreased expression of these genes under manganese stress may indicate that excessive manganese inhibited these cell functions, reduced the rate of glycolysis, and impaired cell energy metabolism, thereby affecting all biosynthesis processes that require energy and carbon skeletons, including amino acid synthesis. *Tkt* plays a pivotal role in the pentose phosphate pathway (PPP), which is involved in the production of ribose-5-phosphate and erythrose-4-phosphate. PPP plays a bifunctional role by furnishing NADPH for oxidative stress defense while simultaneously providing ribose for nucleic acid synthesis, and erythrose-4-phosphate for the synthesis of aromatic amino acids (phenylalanine, tyrosine, tryptophan) [60]. Therefore, in this study, the decrease in *tkt* expression caused by manganese stress directly affected the biosynthesis pathway of aromatic amino acids, and may reduce the antioxidant capacity of cells by reducing the production of NADPH, making cells more susceptible to manganese-induced oxidative damage [61,62]. *Sds* is an enzyme that converts serine to pyruvate and ammonia. Serine is the precursor of a variety of biological molecules (including glycine, cysteine, etc.), and pyruvate is one of the end products of glycolysis, which could enter the tricarboxylic acid cycle or serve as a precursor for the synthesis of other amino acids [63]. The decrease in *sds* expression caused by manganese stress may mean that the decomposition of serine was reduced, or the production of pyruvate was blocked, thus affecting the biosynthesis pathway of pyruvate-related amino acids (such as alanine, valine, leucine, and isoleucine). Taken together, manganese stress interfered with glycolysis and amino acid synthesis in the gill tissue of *Coilia nasus* through multiple mechanisms. These metabolic disorders worked together to damage the energy homeostasis and protein synthesis ability of the gill tissue, which may ultimately affect the growth performance and environmental adaptability of *Coilia nasus*.

## 5. Conclusions

**Figure 10:**
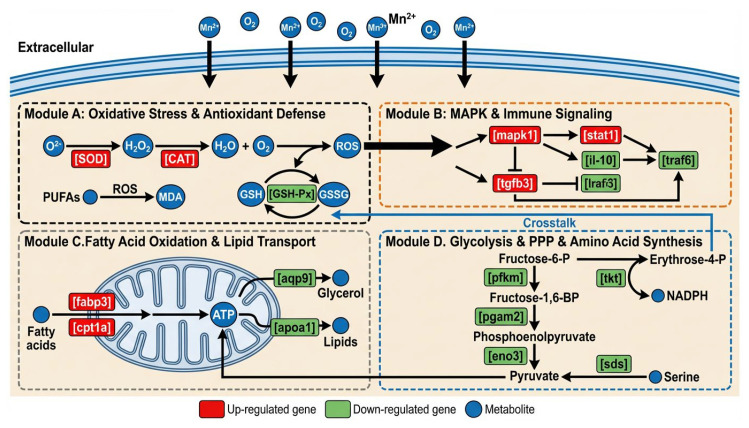
Changes in antioxidant, immune, lipid metabolism, glycolysis and amino acid synthesis pathways in gill tissues of *Coilia nasus* under acute manganese stress.

Transcriptome analysis demonstrated that acute manganese stress profoundly impaired *Coilia nasus* by dysregulating immune- and metabolism-related genes. This dysregulation, driven by manganese-induced oxidative damage, resulted in immunosuppression, metabolic dysfunction, and energy deficit. The specific damage mechanism was shown in Figure 10. The potent toxicity of waterborne manganese enrichment thus critically compromises physiological integrity and may lead to mortality.

## Figures and Tables

**Table 1:** Gill parameters related to antioxidant capacity.

Index	Measurement Methods	Note
SOD	WST-1 method	Assay kits purchased from Jian Cheng Bioengineering Institute (Nanjing, China); Spectrophotometer (Thermo Fisher Multiskan GO, Shanghai, China).
CAT	Ammonium molybdenum acid method	
MDA	TBA method	
GSH	Microplate method	
GSH-Px	Colorimetric method	

**Table 2:** Primers used in the experiment.

Gene Name	Gene Bank/Reference		Primer Sequence (5′-3′)	Product Length
*β-actin*	[26]	Forward	AACGGATCCGGTATGTGCAAAGC	NA
		Reverse	GGGTCAGGATACCTCTCTTGCTCTG	
*traf6*	XP_041967185.1	Forward	AACAGTGCAGCGGTGTTTTC	87
		Reverse	ATCACCGACTGATTGGCTCTG	
*mapk1*	KAG9349995.1	Forward	ACATCATCGGCATCAACGAC	88
		Reverse	ACACACTCACACACACACAC	
*il-10*	XP_012687424.1	Forward	TGTGTCTTTGTGGAGAACGC	97
		Reverse	TCTCCAGGTCGTCATTCTCTTC	
*tgfb3*	XP_041951915.1	Forward	AAATGCCAGCAGCTTGTTCC	147
		Reverse	TTTTTGCCGTCGATGTAGCG	
*stat1*	NC_045153.1	Forward	CACACACTGTGAGTTTGCCG	98
		Reverse	CCGGTAGTGAGGAGGGGTTA	
*pfkm*	XP_041953868.1	Forward	AACACGCCAAACTCTGCATG	122
		Reverse	AGCCACCACTGTGTTTTTGG	
*pgam2*	XP_004538376.1	Forward	AGAGTGCATGGAACCAGGAG	129
		Reverse	AGCACACGTCGAACTTCATG	
*eno3*	XP_012690312.1	Forward	TTATGCTTGAGCTGGATGGC	81
		Reverse	TGCACACAGCCAGAGAAATG	
*pkm*	KAG5273734.1	Forward	TTTGAGGAGCTGCGTCGTAC	129
		Reverse	AGACCTGCCAGATTTGGTGAG	
*fabp3*	XP_034561453.1	Forward	GGCCGAAAAGTTTGCTGGAA	86
		Reverse	TAGCGAAGCCAACACCCATT	
*cpt1a*	XP_041964596.1	Forward	TGGCCATCATCTTCGTCATG	84
		Reverse	TTATGTGTCCATGGCGGTTG	
*aqp9*	XP_041915748.1	Forward	AAGACCCACAGTGGCAGATG	251
		Reverse	GTAAATAGCAGCACGCAGCC	
*apoa1*	XP_041920424.1	Forward	AAGTTGTGGCACTTGCACTG	142
		Reverse	TTCTGTGCAGTCTCCTTCACC	
*tkt*	XP_041952705.1	Forward	AGAGCAACATCAACCTGTGC	146
		Reverse	TTCAGTTGACACGCCATCAC	
*sds*	XP_012687643.2	Forward	ATGAAGCTAGTGGGAGAGCAC	86
		Reverse	TCGTCAACGAACTTGTCAGC	

*traf6*, tumor necrosis factor receptor associated factor 6; *tgfb3*, transforming growth factor beta 3; *mapk1*, mitogen-activated protein kinase 1; *stat1*, signal transducer and activator of transcription 1; *pfkm*, phosphofructokinase muscle; *pgam2*, phosphoglycerate mutase 2; *il-10*, interleukin-10; *eno3*, enolase 3; *pkm*, pyruvate kinase muscle; *fabp3*, fatty acid binding protein 3; *cpt1a*, carnitine palmitoyltransferase 1a; *aqp9*, aquaporin 9; *apoa1*, apolipoprotein a-I; *tkt*, transketolase; *sds*, serine dehydratase. NA, not applicable.

**Table 3:** Summary of Illumina RNA-Seq data.

Sample	Raw Reads	Raw Data (bp)	Clean Reads	Clean Data (bp)	Q20 (%)	Q30 (%)	GC (%)	Unique Mapped (%)	Multiple_Mapped (%)	Total_Mapped (%)
Control-1	40,817,194	6,122,579,100	40,525,862	6,058,094,357	97.56%	93.46%	48.63%	32,072,812 (79.18%)	1,608,833 (3.97%)	33,681,645 (83.15%)
Control-2	42,403,780	6,360,567,000	42,131,230	6,295,325,718	97.57%	93.40%	49.21%	34,726,031 (82.46%)	1,231,206 (2.92%)	35,957,237 (85.38%)
Control-3	42,826,516	6,423,977,400	42,531,126	6,354,173,246	97.53%	93.14%	49.08%	34,123,531 (80.26%)	1,467,926 (3.45%)	35,591,457 (83.71%)
Mn-1	46,706,414	7,005,962,100	46,405,000	6,938,742,006	97.65%	93.72%	49.16%	38,514,876 (83.02%)	1,633,563 (3.52%)	40,148,439 (86.54%)
Mn-2	51,826,938	7,774,040,700	51,456,056	7,691,585,772	97.39%	93.15%	48.86%	42,335,940 (82.30%)	1,813,540 (3.53%)	44,149,480 (85.83%)
Mn-3	42,741,558	6,411,233,700	42,459,864	6,343,233,332	97.46%	93.16%	48.28%	34,565,657 (81.44%)	1,203,784 (2.84%)	35,769,441 (84.27%)

## Data Availability

Data are contained within the article.

## Supplementary Materials

The following supporting information can be downloaded at: https://www.mdpi.com/article/10.3390/ani16060974/s1, Table S1: The mortality rate of acute stress.
