# Supplementary material for: Transcriptome-Based Analysis of the Mechanism of Acute Manganese-Induced Immune Function Decline and Metabolic Disorders in Estuarine Tapertail Anchovy (Coilia nasus)
Source: Animals (Basel). 2026 Mar 20;16(6):974. doi: 10.3390/ani16060974 (PMC13023277; doi:10.3390/ani16060974)
Supplement: Supplementary file 1 [file animals-16-00974-s001.zip › animals-4126088-supplementary.pdf]

Table S1. The mortality rate of acute stress

Groups	Acute Stress Times					
	0h	4h	8h	12h	16h	20h	24h
Control Group	0.00±0.00	0.00±0.00	0.00±0.00	0.00±0.00	0.00±0.00	0.00±0.00	0.00±0.00
PD1	0.00±0.00	1.67±2.89	8.33±5.77	13.33±2.89	16.67±2.89	18.33±2.89	26.67±2.89
PD2	0.00±0.00	5.00±0.00	13.33±5.77	30.00±5.00	38.33±2.89	66.67±7.64	80.00±10.00
PD3	0.00±0.00	6.67±2.89	15.00±5.00	35.00±5.00	40.00±8.66	73.33±7.64	90.00±5.00
PD4	0.00±0.00	10.00±5.00	20.00±5.00	45.00±5.00	68.33±2.89	91.67±2.89	100.00±0.00
PD5	0.00±0.00	15.00±0.00	38.33±7.64	70.00±5.00	91.67±2.89	100.00±0.00	100.00±0.00
PD6	0.00±0.00	25.00±0.00	66.67±10.41	100.00±0.00	100.00±0.00	100.00±0.00	100.00±0.00
